# Neurological Manifestations and Complications of the Central Nervous System as Risk Factors and Predictors of Mortality in Patients Hospitalized with COVID-19: A Cohort Study

**DOI:** 10.3390/jcm12124065

**Published:** 2023-06-15

**Authors:** Ana Luisa Corona-Nakamura, Martha Judith Arias-Merino, Rayo Morfín-Otero, Guillermo Rodriguez-Zavala, Alfredo León-Gil, Juan Ramsés Camarillo-Escalera, Idarmis Brisseida Reyes-Cortés, María Gisela Valdovinos-Ortega, Erick René Nava-Escobar, Ana María de la Paz Villaseñor-Corona, Mario Alberto Mireles-Ramírez, Aldo Guadalupe Cisneros-Aréchiga, Ofelia Padilla-De la Torre, Héctor Raúl Pérez-Gómez, Eduardo Rodríguez-Noriega

**Affiliations:** 1 High Specialty Medical Unit, Western National Medical Center of the Mexican Institute of Social Security, Guadalajara 44340, Mexico; 2 Western Clinical Research Institute, Zapopan 45030, Mexico; 3 Institute of Infectious and Experimental Pathology, University Center of Health Sciences, University of Guadalajara, Guadalajara 44280, Mexico

**Keywords:** central nervous system, COVID-19, risk factors, predictors of mortality

## Abstract

The aim of this study was to analyze the risk factors and predictors of mortality in a retrospective cohort of patients with coronavirus disease (COVID-19) who presented central nervous system (CNS) manifestations and complications when admitted to hospital. Patients hospitalized from 2020 to 2022 were selected. Demographic variables; history of neurological, cardiological and pulmonary manifestations; comorbidities; prognostic severity scales; and laboratory tests were included. Univariate and adjusted analyses were performed to determine risk factors and predictors of mortality. A forest plot diagram was used to show the strength of the associated risk factors. The cohort included 991 patients; at admission, 463 patients presented CNS damage and of these, 96 hospitalized patients presented de novo CNS manifestations and complications. We estimate a general mortality of 43.7% (433/991) and 77.1% (74/96), for hospitalized patients with de novo CNS manifestations and complications, respectively. The following were identified as risks for the development of hospital CNS manifestations and complications when in hospital: an age of ≥64 years, a history of neurological disease, de novo deep vein thrombosis, D-dimer ≥ 1000 ng/dL, a SOFA ≥ 5, and a CORADS 6. In a multivariable analysis, the mortality predictors were an age of ≥64 years, a SOFA ≥ 5, D-dimer ≥ 1000 ng/mL and hospital CNS manifestations and complications when admitted to hospital. Old age, being hospitalized in critical condition, and having CNS manifestations and complications in hospital are predictors of mortality in hospitalized patients with COVID-19.

## 1. Introduction

At the end of 2019, an emerging viral infectious pathology called COVID-19 emerged, caused by the severe acute respiratory syndrome virus 2 (SARS-CoV-2) [1]. Globally, as of 15 January 2023, a total of 671,314,125 confirmed cases of COVID-19 and 6,730,278 deaths have been reported, with a recovery rate of about 96%. In Mexico, as of that same date, a total of 7,309,154 cases and 331,510 deaths have been reported, with a recovery rate of 89% (ranking 19th) (https://www.worldometers.info/coronavirus/, accesed on 16 October 2022) [2].

It has been reported that up to 80% of patients hospitalized with COVID-19 present some neurological manifestation; moreover, manifestations and complications specifically of the CNS (mainly strokes) have been observed as a predictive factor for mortality [3]. Battaglini et al. [4] in particular cases, reported a mortality of up to 92% (stroke and ischemic-hypoxic brain damage).

SARS-CoV-2 has been shown to enter directly through the olfactory nerves where the axons connect to different regions of the CNS [5]. In hematological dissemination, the virus reaches the brain and infects the endothelial cells of the blood–brain barrier (BBB) and of the cerebrospinal fluid in the choroid plexus. The three mechanisms for dissemination through the BBB are: (1) the Trojan horse pattern, where the virus uses leukocytes and myeloid cells to enter the CNS; (2) paracellular migration, where the virus crosses the BBB by destroying the Trojan horse complex; and (3) in the vascular endothelium of the BBB, where SARS-CoV-2 binds to the human receptor for angiotensin-converting enzyme 2 (ACE-2). In addition to the mechanisms of invasion of the CNS, there are other neuropathogenic mechanisms such as hypoxic brain injury and immunological injury mediated by a cytokine storm. The latter leads to rupture of the BBB, manifesting in elevations in interleukin 6 (IL-6), D-dimer, C-reactive protein (CRP), and lymphopenia inflammatory markers [3,6,7].

The main hospital neurological manifestations of the CNS in patients with COVID-19 that have been reported are delirium, hallucinations, drowsiness, stupor, dysarthria, aphasia, hemiparesis, hemiplegia, anisocoria, and seizures, and complications such as ischemic stroke, hemorrhagic stroke, subarachnoid hemorrhage, encephalitis, epilepsy NOVO, ataxia, and others have been reported [4,8,9,10]. A series of studies have been published establishing associations between neurological manifestations and complications of the CNS and mortality [1,4,8]. In our study, we had the opportunity to perform an adjusted odds ratio analysis to determine predictors of mortality in our cohort of COVID-19 patients during hospital evolution, which allowed us to emphasize the role of neurological manifestations and complications of the CNS de novo. Therefore, the objective of this study was to analyze the risk factors and predictors of mortality in a retrospective cohort of patients with COVID-19 who presented CNS manifestations and complications when admitted to hospital.

## 2. Materials and Methods

### 2.1. Participants and Procedures

**Figure 1:**
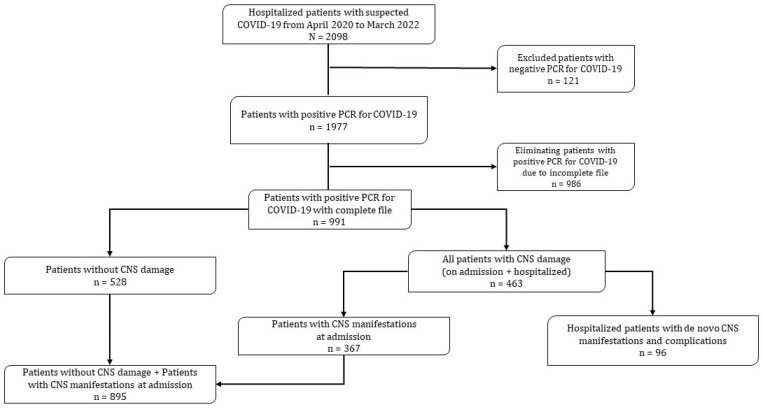
Flow diagram. CNS, central nervous system.

This study was performed on a retrospective cohort and it was carried out in a medical and surgical specialty hospital in western Mexico. Of 1977 hospitalized patients (April 2020 to March 2022) with a confirmed diagnosis of COVID-19 by PCR, 991 complete medical records were reviewed. Of the 463 patients who presented with CNS neurological damage (any neurological signs or symptoms) during admission and/or during hospitalization, 96 hospitalized patients with de novo (recently presented or newly diagnosed) CNS manifestations and complications (neurological cohort) were selected for further analysis (Figure 1). The study cohort includes patients ≥18 years of age and of both genders.

The study variables included were demographic data, comorbidities, previous history of neurological manifestations, cardiopulmonary manifestations, history of pulmonary manifestations, and the time of onset of general and respiratory symptoms.

On admission, the patients were assessed in the emergency department and/or in the hospital area through the protocol established in the hospital that includes a SOFA ≥ 5 (Sepsis Related Organ Failure Assessment), a NEWS2 (National Early Warning Score), a CORADS 6, and also, severe acute respiratory distress syndrome (ARDS) (PaO_2_/FiO_2_ ≤ 100 mmHg) and laboratory test results showing D-dimer ≥ 1000 ng/mL, troponin, B-type natriuretic peptide (BNP), thrombocytopenia ≤ 150 cells (10^3^/µL) and lymphopenia ≤ 900 cells (10^3^/µL) (including cerebrospinal fluid). Assessment was supported with images such as computed tomography and/or magnetic resonance images with and without gadolinium of the skull and/or electroencephalograms.

A neurological diagnosis of patients admitted to hospital (with CNS manifestations and complications of CNS, neurological cohort) was determined by neurology and neurosurgery specialists.

The following were considered neurological manifestations of the CNS:

- At admission: headache and vomiting.
- At hospital: delirium, hallucinations, altered state of consciousness (drowsiness and stupor), language disorders (dysarthria and aphasia), motor deficits (hemiparesis, hemiplegia and quadriparesis), anisocoria, and seizures.

Likewise, the following neurological complications of the CNS were considered:

- At hospital: ischemic stroke (left and right middle cerebral artery; right and left carotid artery), hemorrhagic stroke, subarachnoid hemorrhage, encephalitis, de novo epilepsy, and ataxia (uncoordinated movements).

The following were considered neurological manifestations of the PNS: myalgias, anosmia, and dysgeusia (disturbances of taste and smell) at admission; and cranial neuropathy, Guillain Barré syndrome and myopathy at hospitalization. Other extraneurological complications were also analyzed, such as hematological and cardiovascular complications (pulmonary thromboembolism, disseminated intravenous coagulation, deep venous thrombosis, de novo congestive heart failure and acute myocardial infarction, among others).

Patients were monitored from admission to discharge or outcome (improvement or death).

### 2.2. Statistical Analysis

Quantitative variables were presented through medians and interquartile ranges (IQR) and nominal variables in percentage. To compare the differences between the variables (including risk factors and mortality predictors), a Mann–Whitney U test, an X2 test, or a Fisher’s exact test were used. Univariate analysis was performed between the variables included in the cohort of patients with and without de novo CNS neurological manifestations and/or complications Univariate analyses and multivariate logistic regression analyses were performed to estimate adjusted odds ratios and determine risk factors and predictors of mortality, respectively. A *p*-value of <0.05 (two tails) was considered statistically significant. An adjusted odds ratio model was designed with the variables that were statistically significant in the univariate analysis of mortality. Additionally, a forest plot diagram was used to show the risk factors associated with the presentation of neurological manifestations and complications in the CNS observed in hospitalized patients with COVID-19. SPSS V25^®^ (IBM, Armonk, NY, USA) and RevMan version 5.3 software were used for statistical analyses.

## 3. Results

Of the 991 complete records reviewed, we found 614 male patients (62.0%) and 377 females (38.0%) with a median age of 62 years (IQR 50–71). Of these, a total of 96 hospitalized patients with de novo CNS manifestations and complications were found; 57 patients (59.4%) were men and 39 (40.6%) were women, with an age range of 19–91 years of age and a median age of 70 (IQR 62–77).

Table 1 describes the frequency of presentation of the neurological study variables (CNS and PNS manifestations and complications) during admission and the evolution of the hospitalized patients included in the COVID-19 cohort.

At admission, headache was observed as the main neurological manifestation, and de novo CNS manifestations observed in hospitalized patients were delirium, drowsiness, and stupor. Among the de novo CNS complications, stroke and ischemic stroke were most frequently observed. The most frequent neurological manifestations in the peripheral nervous system (PNS) on admission were myalgia, anosmia, and dysgeusia. The main neurological complications were cranial neuropathy, de novo Guillain Barré syndrome and myopathy. Twenty patients had concomitant manifestations or complications of the central and peripheral nervous system (stroke and dysgeusia, delirium and dysgeusia) (Table 1).

At admission, among the comorbidities presented by the patients that made up the study cohort, we found the following in descending order of frequency: arterial hypertension (549 patients, 55.4%), diabetes mellitus (412, 41.6%), obesity (396/814, 48.6%), smoking (221/982, 25.5%), chronic kidney disease (100, 10.1%), history of any pulmonary disease (76, 7.7%), previous coronary artery disease (55, 5.5%), chronic obstructive pulmonary disease (COPD) (50, 5.0%), malignancy (30, 3.0%), and any neurological disease (30, 3.0%). The following comorbidities were present in less than 3.0% of patients: history of heart failure, steroid use, asthma, history of previous stroke, chronic liver disease, kidney transplant, previous peripheral vascular event, history of epilepsy, infection by human immunodeficiency virus (HIV), and multiple sclerosis (Table 2).

### 3.1. Risk Factors Associated with Hospital CNS Manifestations and Complications in Patients with COVID-19

In the univariate analysis, we found the following as statistically significant risk factors: age ≥ 64 years (OR 2.60, CI 1.67–4.06, *p* = 0.000), arterial hypertension (OR 1.98, CI 1.26–3.12, *p* = 0.003), diabetes mellitus (OR 1.76, CI 1.15–2.68, *p* = 0.008), chronic liver disease (OR 5.52, CI 1.58–19.20, *p* = 0.016), history of any neurological disease (OR 5.09, CI 2.31–11.22, *p* = 0.000), history of stroke (OR 3.66, CI 1.40–9.59, *p* = 0.005), history of heart failure (OR 3.27, CI 1.35–7.91, *p* = 0.005), history of epilepsy (OR 9.59, CI 1.91–48.20, *p* = 0.014), and history of COPD (OR 2.15, CI 1.01–4.58, *p* = 0.041) (Table 2).

Among the laboratory findings, the following risk factors were found: lymphopenia (≤0.900 10^3^ cells/µL) (OR 1.90, IC 1.24–2.90, *p* = 0.003), platelets (≤150 10^3^ cells/µL) (OR 2.98, IC 1.84–4.84, *p* = 0.000), D-dimer (≥1000 ng/mL) (OR 3.87, CI 2.38–6.27, *p* = 0.000), troponin ng/mL, median 27.5 (IQR 11.3–27.5, *p* = 0.000), and B-type natriuretic peptide pg/mL, median 1858 (IQR 410–6216, *p* = 0.000) (Table 2).

Table 3 presents a description and univariate analysis of the evolution of the patients during admission and their hospital stay and the outcomes.

Admission. A median of 7 days (IQR 5–11 days) from COVID symptom onset to hospital admission was calculated for the 991 patients.

In assessing the severity of the disease on admission associated with neurological manifestations and complications in the CNS through study protocols, we found the following prognostic scales to be statistically significant in these patients: CORADS 6 (OR 3.46, CI 2.10–5.69, *p* = 0.000), severe ARDS (OR 2.07, CI 1.34–3.20, *p* = 0.001), SOFA ≥ 5 (OR 5.48, CI 3.23–9.29, *p* = 0.000), and NEWS2 score ≥ 8 (OR 2.59, CI 1.63–4.12, *p* = 0.004).

The range in duration of the hospital stay for all patients was 1–53 days, with a median of 10 days (IQR 6–17). The neurological cohort presented various extraneurological complications, among the most important were de novo cardiovascular manifestations (OR 3.98, CI 1.97–8.04, *p* = 0.000), de novo congestive heart failure (OR 5.34, CI 2.08–13.72, *p* = 0.000), and de novo pulmonary thromboembolism (OR 5.74, CI 1.35–24.41, *p* = 0.034). Among the hematological complications, de novo deep vein thrombosis was found to be significant (OR 11.87, CI 3.55–39.65, *p* = 0.000).

Outcomes. In the patients included in the study cohort, there were 433/991 deaths (43.7%). Of the 96 hospitalized patients in the neurological cohort with de novo CNS manifestations and complications, 74 people (77.1%) had a fatal outcome (OR 5.02, CI 3.06–8.23, *p* = 0.000); 73 of these deaths occurred within 29 days. For patients in the neurological cohort, the risks of requiring invasive mechanical ventilation (OR 2.09, CI 1.36–3.20, *p* = 0.001) and of presenting septic shock (OR 2.77, CI 1.80–4.25, *p* = 0.000) were statistically significant (Table 3).

**Figure 2:**
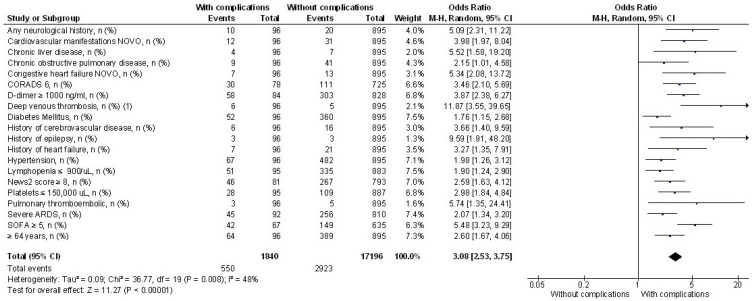
Forest plot of risk factors associated with CNS manifestations and complications in patients hospitalized with COVID-19.

Figure 2 presents a diagram of the most relevant results of the univariate analysis of the risk factors associated with CNS manifestations and complications in patients hospitalized with COVID-19. The result of the test for heterogeneity was calculated as I^2^ = 48%. The test for overall effect was OR 3.08, 95% CI 2.53–3.75, Z = 1, *p* < 0.00001.

### 3.2. Mortality Predictors Associated with Hospital CNS Manifestations and Complications in Patients with COVID-19

In the univariate analysis, age, male sex and the following comorbidities were found to be risk factors for mortality: arterial hypertension, diabetes mellitus, chronic kidney disease, any pulmonary history, COPD, and history of heart failure. Additionally, de novo CNS manifestations and complications present during hospitalization were a risk factor of mortality (OR 5.02, IC 3.06–8.23, *p* = 0.000), with delirium, drowsiness, stupor, stroke (OR 7.10, IC 2.06–24.52, *p* = 0.001), and ischemic stroke (OR 4.82, CI 1.34–17.39, *p* = 0.012) the most significant.

Laboratory findings that were consistent with an increased risk of mortality were lymphopenia ≤ 0.900 10^3^ cells/µL, platelets ≤ 150 10^3^ cells/µL and D-dimer ≥ 1000 ng/mL. In the severity scales, CORADS 6, Severe ARDS, SOFA ≥ 5 and NEWS2 ≥ 8 were also observed as risk factors of mortality for patients with COVID-19.

A multivariate logistic regression analysis was designed selecting the following variables: age of 64 years, sex, diabetes mellitus, arterial hypertension, any pulmonary history, general neurologic (CNS + PNS) manifestations and complications, hospital CNS manifestations, and complications, D-dimer ≥ 1000 ng/mL, lymphopenia ≤ 0.900 10^3^ cells/µL, and a SOFA ≥ 5.

When adjusting the ORs, the following were observed as predictors of mortality: age ≥ 64 years (OR 2.87, CI 2.02–4.08, *p* = 0.000), SOFA ≥5 (OR 4.52, CI 2.93–6.99, *p* = 0.000), D-dimer ≥ 1000 ng/mL (OR 2.25, CI 1.57–3.23, *p* = 0.000). and hospital CNS manifestations and complications (OR 3.05, CI 1.39–6.72, *p* = 0.006) (Table 4).

## 4. Discussion

The mortality of patients without de novo CNS neurological manifestations and complications was 40.1% (359/895), and in patients with de novo CNS neurological manifestations and complications, mortality was higher, 77.1% (74/96). The above shows the role of a compromised neurological system in the outcome of patients hospitalized with COVID-19 and expresses the mortality predictors found in this study, such as older age, de novo CNS manifestations and complications, D-dimer, and SOFA ≥ 5, in analytical terms.

Among the extrapulmonary manifestations and complications observed in patients with COVID-19, neurological manifestations of the central nervous system had a great impact due to their risk of high mortality [3,4,11,12].

Risk factors and predictors of mortality were analyzed in a cohort of patients with COVID-19 who presented with de novo hospital neurological manifestations and complications, using adjusted statistical analysis models. A forest plot diagram was also used to show the strength of the associated risk factors.

Given the low mortality in patients with PNS manifestations and complications (10.6%), we considered analyzing patients with de novo CNS manifestations and complications separately. Through the analysis strategy, we were able to identify the risks and predictors of mortality in the neurological cohort, whose manifestations and complications were severe and that resulted in a poor prognosis.

The most frequent neurological complication associated with mortality in our study was stroke (OR 7.09, CI 2.06–24.52, *p* = 0.001)—particularly, ischemic stroke. Hingorani et al., (2022) reported that this cerebrovascular event is associated with older age, comorbidities and critical illness, with mortality being five times higher than in patients with stroke not infected by COVID-19. In addition to the fact that acute ischemic stroke tends to be more severe, its predominant etiology is associated with large vessel occlusion and cardioembolic events.

Among extraneurological complications, de novo cardiovascular complications (mainly, myocardial infarction, heart failure, pulmonary thromboembolism, and cardiac arrhythmias) were observed as a risk factor associated with mortality in the patients included in our study.

The study cohort was represented by a greater number of men than women, with a median age of 62 years. This distribution of patients was similar to that reported in other studies [8,12,13,14]. The cohort presenting with neurological CNS symptoms while in hospital also had a greater number of men than women, but with a higher mean age in this study (70 years).

The chronic degenerative diseases that occurred most frequently in the general study cohort were, as in other reviewed reports, arterial hypertension, diabetes mellitus, COPD, history of heart failure, and history of any neurological disease, mainly cerebrovascular diseases [14,15,16].

The following were classified as risk factors in hospitalized patients with de novo CNS manifestations and complications: age ≥ 64 years, arterial hypertension, diabetes mellitus, history of any neurological disease, history of stroke, history of heart failure, history of epilepsy, and a history of COPD. These have also been reported in other studies as risk factors [7,8,14].

Lymphopenia, thrombocytopenia, and elevated levels of D-dimer, troponin, and B-type natriuretic peptide were among the risk factors in the hospital neurological cohort among the laboratory findings, as reported in previously reviewed studies [4,7,14,16].

Additionally, in the univariate analysis, the following severity scale results, assessed during the admission of the patients and commonly used in our hospital for severity assessment in patients with COVID-19, were found as risk factors: a CORADS score of 6, severe ARDS, SOFA ≥ 5, and a NEWS2 score ≥ 8. In a study on mortality by Na et al. [17], they reported the use and factors associated with mortality of older adult patients with COVID-19 with a univariate analysis and Cox regression through the SOFA, CURB-65 score, and MEWS (modified early warning score) severity scales. The study was, however, applied to overall mortality from COVID-19. Flores-Silva et al. [18] reported the statistically significant effect of the CALL score and NEWS2 score assessed on admission in patients with COVID-19 analyzed through a semiquantitative score (low, medium and high risk) in a univariate analysis of the presentation of neurological signs and symptoms. These data indicate the usefulness of these scales in the management of COVID-19.

It has been reported that myocardial ischemia may predispose patients to further stroke damage as an extraneurological complication associated with CNS complications in patients with COVID-19 [3]. It has also been reported that the risk of presenting arterial and venous thrombosis is increased, favoring the occurrence of an ischemic stroke, even without a history of vascular disease. This occurrence is also associated with elevated levels of D-dimer and troponin [19].

The general cohort of patients included in our study presented a mortality of 43.7%, well above different rates reported worldwide; for example, in the Republic of Korea, the mortality of an elderly cohort of patients in hospitals was estimated at 25.5% [17]. However, patients with neurological manifestations and/or complications of the CNS while in hospital registered worse outcomes in our study among patients with COVID-19 (mortality of 77.1%).

The mortality rate in patients with COVID-19 due to respiratory complications ranges in different countries from 13 to 73%, since mortality varies from one country to another due to the age of patients and the level of access to treatment [1].

It is difficult to compare hospital mortality because the cohorts in the published studies refer to different stages of the COVID-19 pandemic in relation to time, different places, and different levels of access to vaccination. In addition, the different hospital conditions due to installed capacity, the different number of beds, the availability of human and material resources, etc., together with the great sociodemographic inequalities in the population and between the different hospitals, further complicates the comparison of said mortality. In a second-level hospital in Mexico City, a general hospital mortality from COVID-19 of 68.3% was observed in a retrospective cohort. The authors explain that this high mortality is partly justified by the low socioeconomic conditions of their custom population and the great inequities in hospital resources in the area of said hospital [20].

It must be considered, on the one hand, that our hospital regularly admits patients with severe complications referred from second-level hospitals and from the suburban and rural areas of Western Mexico. On the other hand, the low levels of prior vaccination in admitted patients should also be noted. Including a forest plot diagram allowed us to evaluate the findings of greatest interest and improve the precision of estimating risks. The diamond in the diagram is clearly to the right of the reference line, with a narrow confidence interval that allows one to determine the precision of the risk estimate and that the association studied was not due to chance [21].

Another one of the main strengths of the present study was the inclusion of just over 280 variables during the analysis of admission, hospitalization, and mortality.

Through a multivariate logistic regression analysis, it was found that an age of ≥64 years, hospital de novo CNS manifestations and complications while in hospital, D-dimer ≥ 1000 ng/mL and a SOFA ≥ 5 were predictors of mortality. In the reviewed literature, the works that present regression models to calculate adjusted odds ratios present results of general hospital mortality from COVID-19 and of patients with neurological manifestations and complications jointly from both the PNS and the CNS and other approaches with different designs [8,17].

Of the demographic variables, advanced age has been reported in different studies as a risk factor and/or predictor of mortality for COVID-19 patients and COVID-19 cohorts presenting with neurological damage, as determined in the present work [3,8,17,22].

The fact that the neurological evaluation was carried out by neurologists and neurosurgeons may contribute to reducing misclassifications of the data on neurological manifestations and complications, in addition to reducing the difficulties in discriminating the effects of aging in our patient cohort, which included elderly patients who have chronic degenerative diseases.

Battaglini et al. [4], specify that the patients who presented with more severe COVID-19 had greater CNS involvement (particularly patients with stroke) and higher D-dimer levels in their study on neurological manifestations in patients with SARS-CoV-2 infection.

In Na et al.’s [17] study on mortality predictors, they specify the utility of the SOFA score, where the highest score was associated with the highest mortality rate in older adult patients with severe COVID-19, which was also found to be statistically significant in the present study.

The SOFA score has been shown to be an important tool in the evaluation of critically ill patients, since it includes clinical, physiological and laboratory parameters that assess pulmonary, hematological, neurological, renal, hepatic, and cardiovascular functions. However, additional study is required to examine its scope in the patients included in this study.

In a review article [3], when analyzing the neurological manifestations and complications of the CNS of patients with COVID-19, the authors explained that stroke has been systematically associated with more serious and fatal outcomes, estimating that hospital mortality could be 5-times higher in COVID-19 patients with de novo CNS neurological complications. Furthermore, they reported that the pathophysiology of these neurological events can be explained by virally mediated hypercoagulability, cytokine storm, cardiac effects and/or cerebrovascular arteriopathy. Additionally, in a study by Battaglini et al. [4], the authors concluded that patients who suffered a stroke have a worse prognosis.

## 5. Limitations

In the multivariate logistic regression analysis on the predictors of mortality, the variables of invasive mechanical ventilation and an admission to the ICU were excluded due to the indications of the triage protocol for the admission and hospitalization of patients with COVID-19 at the critical point of hospital saturation. In addition, many of the same patients rejected intubation for cultural reasons.

Other limitations of the study included the retrospective and longitudinal nature of this work, in addition to the health emergency which restricted the availability of data for excluded patients.

The present study considers patients from a one hospital, so it was limited in relation to the human and material resources available during the COVID-19 pandemic. This also limited the number of subjects that could be included in the cohort; however, the fact that this study only considers one hospital allowed us to better systematize and unify the data collected.

The authors of this article, like all health professionals around the world, had to face this serious emerging disease without knowing its natural history, diagnosis, prognosis. and treatment. We present this publication to allow the reader to refer to data on our specific patient population, including data on hospital mortality from COVID-19, among other characteristics.

## 6. Conclusions

Old age, being admitted in a critical condition and presenting comorbidities of chronic degenerative diseases, as well as having hospital manifestations and complications of the CNS, are determining factors in the prediction of mortality in patients hospitalized with COVID-19 as observed in the present study.

It is important to regularly update specific strategies for patients with COVID-19 who have risk factors for developing neurological complications mainly of the CNS to understand the pathophysiology of these complications and to detect cerebrovascular alterations earlier, thus avoiding a poor prognosis.

## Figures and Tables

**Table 1:** Frequency of neurological manifestations and complications at admission and while in hospital in the cohort of COVID-19 patients.

Study Variables	All Patients (n = 991)	Without Neurological Complications in the CNS n = 895 (90.3%)	Neurological Complications in the CNS n = 96 (9.7%)
Neurological manifestations in the central nervous system (CNS) on admission			
Headache, n (%)	392 (39.6)	350 (39.1)	42 (43.8)
Vomiting, n (%)	31/641 (4.87)	25/553 (4.5)	6/88 (6.8)
Neurological manifestations in the central nervous system (CNS) while in hospital			
Delirium, n (%)	63 (6.4)	-	63 (65.6)
Hallucinations, n (%)	4 (0.4)	-	4 (4.2)
Drowsiness, n (%)	32/990 (3.2)	-	32 (33.3)
Stupor, n (%)	21 (2.1)	-	21 (21.9)
Dysarthria, n (%)	5 (0.5)	-	5 (5.2)
Aphasia, n (%)	5 (0.5)	-	5 (5.2)
Hemiparesis, n (%)	9 (0.9)	-	9 (9.4)
Hemiplegia, n (%)	4 (0.4)	-	4 (4.2)
Quadriparesis, n (%)	3 (0.3)		3 (3.1)
Anisocoria, n (%)	4 (0.4)	-	4 (4.2)
Seizures, n (%)	9/990 (0.9)	-	9 (9.4)
Hospital neurological complications in the central nervous system (CNS) while in hospital			
Stroke, n (%)	19 (1.9)	-	19 (19.8)
Ischemic stroke, n (%)	14 (1.4)	-	14 (14.6)
Ischemic in the left middle cerebral artery, n (%)	7 (0.7)	-	7 (7.3)
Ischemic in right middle cerebral artery, n (%)	5 (0.5)	-	5 (5.2)
Left carotid ischemic stroke, n (%)	1 (0.1)	-	1 (1.0)
Right carotid ischemic stroke, n (%)	1 (0.1)	-	1 (1.0)
Hemorrhagic stroke, n (%)	5 (0.5)	-	5 (5.2)
Subarachnoid hemorrhage, n (%)	4 (0.4)	-	4 (4.2)
Encephalitis, n (%)	3 (0.3)	-	3 (3.1)
Epilepsy NOVO, n (%)	9 (0.9)	-	9 (9.4)
Ataxia, n (%)	7 (0.7)	-	7 (7.3)
Neurological manifestations in the peripheral nervous system (PNS) on admission			
Myalgias, n (%)	185 (18.7)	168 (18.8)	17 (17.7)
Anosmia, n (%)	82 (8.3)	75 (8.4)	7 (7.3)
Dysgeusia, n (%)	94 (9.5)	86 (9.6)	8 (8.3)
Hospital neurological complications in the peripheral nervous system (PNS) while in hospital			
Cranial neuropathy, n (%)	7 (0.7)	7 (7.3)	-
Guillain Barré syndrome de novo, n (%)	1 (0.10)	1 (0.11)	-
Myopathy, n (%)	7 (0.7)	5 (0.6)	2 (2.1)

**Table 2:** Demographic and laboratory characteristics at admission in COVID-19 patients with hospital CNS manifestations and complications while in hospital.

Demographic Characteristics and Risk Factors	All Patients n = 991	Without De Novo CNS Disorders n = 895 (90.3%)	With De Novo CNS Disorders n = 96 (9.7%)	OR	CI 95%	*p*
Age years, median (IQR) (range)	62 (50–71)	60 (49–70), (20–95)	70 (62–76.8), (19–91)			0.000
≥64 years, n (%)	453 (45.7)	389 (43.5)	64 (66.7)	2.60	1.67–4.06	0.000
Male gender, n (%)	614 (62.0)	557 (62.2)	57 (59.4)			0.583
Unvaccinated patients, n (%)	752/771 (97.5)	679/695 (97.7)	73/76 (96.1)			0.422
Comorbidities						
Hypertension, n (%)	549 (55.4)	482 (53.9)	67 (69.8)	1.98	1.26–3.12	0.003
Diabetes mellitus, n (%)	412 (41.6)	360 (40.2)	52 (54.2)	1.76	1.15–2.68	0.008
Obesity, n (%)	396/814 (48.6)	366/735 (49.8)	30/79 (38.0)			-
Smoking, n (%)	221/982 (22.5)	195/886 (22.0)	26/96 (27.1)			0.258
Chronic kidney disease, n (%)	100 (10.1)	86 (9.6)	14 (14.6)			0.124
Any pulmonary history, n (%)	76 (7.7)	65 (7.3)	11 (11.5)			0.142
Chronic obstructive pulmonary disease, n (%)	50 (5.0)	41 (4.6)	9 (9.4)	2.15	1.01–4.58	0.041
Asthma, n (%)	26 (2.6)	24 (2.7)	2 (2.1)			1.000
History of coronary disease, n (%)	55 (5.5)	51 (5.7)	4 (4.2)			0.813
History of heart failure, n (%)	28 (2.8)	21 (2.3)	7 (7.3)	3.27	1.35–7.91	0.005
History of peripheral vascular event, n (%)	15 (1.5)	12 (1.3)	3 (3.1)			0.171
Malignancy, n (%)	30 (3.0)	25 (2.8)	5 (5.2)			0.202
Any neurological history, n (%)	30 (3.0)	20 (2.2)	10 (10.4)	5.09	2.31–11.22	0.000
History of cerebrovascular disease, n (%)	22 (2.2)	16 (1.8)	6 (6.3)	3.66	1.40–9.59	0. 005
History of epilepsy, n (%)	6 (0.6)	3 (0.3)	3 (3.1)	9.59	1.91–48.20	0.014
Multiple sclerosis, n (%)	2 (0.2)	1 (0.1)	1 (1.0)			0.184
Chronic steroid use, n (%)	23/842 (2.7)	19/754 (2.5)	4/88 (4.5)			0.289
Chronic liver disease, n (%)	11 (1.1)	7 (0.8)	4 (4.2)	5.52	1.58–19.20	0.016
Kidney transplant, (%)	17 (1.7)	17 (1.9)	-			-
HIV infection, n (%)	5 (0.5)	4 (0.4)	1 (1.0)			0.400
Clinical and laboratory findings						
Headache	392 (39.6)	350 (39.1)	42 (43.8)			0.377
Vomiting	31/641 (4.8)	25/553 (4.5)	6/88 (6.8)			0.351
Lymphopenia ≤ 0.900 cells (10^3^/µL), n (%)	386/978 (39.5)	335/883 (37.9)	51/95 (53.7)	1.90	1.24–2.90	0.003
Platelets ≤ 150 cells (10^3^/µL), n (%)	137/982 (14.0)	109/887 (12.3)	28/95 (29.5)	2.98	1.84–4.84	0.000
D-dimer ≥ 1000 ng/mL, n (%)	361/912 (39.6)	303/828 (36.6)	58/84 (69.0)	3.87	2.38–6.27	0.000
Troponin ng/mL, median (IQR)	12.4 (2.9–36.7)	11.4 (2.6–32.4)	27.5 (11.3–27.5)			0.000
BNP pg/mL, median (IQR)	635 (195–2304)	561 (176–1940)	1858 (410–6216)			0.000

COVID-19: coronavirus disease 2019. CNS: central nervous system. IQR: interquartile range. HIV: human immunodeficiency virus. BNP: B-type natriuretic peptide. Ng/mL: nanograms/milliliters. Pg/mL: picograms/milliliters.

**Table 3:** Evolution (admission–inpatient stay–outcomes) in COVID-19 patients with CNS manifestations and complications while in hospital.

Covariates	All Patients n = 991	Without De Novo CNS Disorders n = 895 (90.3%)	With De Novo CNS Disorders n = 96 (9.7%)	OR	CI 95%	*p*
Onset of symptoms at hospital admission (days) median (IQR) (range)	7 (5–11)	7 (5–11) (1–41)	7 (4–10.3) (1–53)			0.172
Severity of illness on admission associated with neurological complications						
CORADS 6, n (%)	141/803 (17.6)	111/725 (15.3)	30/78 (38.5)	3.46	2.10–5.69	0.000
Severe ARDS, n (%) on admission	301/902 (33.4)	256/810 (31.6)	45/92 (48.9)	2.07	1.34–3.20	0.001
CURB-65, median (IQR)	1 (0–2)	1 (0–2)	2 (1–3)			0.000
qSOFA, median (IQR)	1 (1–1)	1 (1–1)	2 (1–3)			0.000
SOFA ≥5, n (%)	191/702 (27.2)	149/635 (23.5)	42/67 (62.7)	5.48	3.23–9.29	0.000
NEWS2 score ≥8, n (%)	313/874 (35.8)	267/793 (33.7)	46/81 (56.8)	2.59	1.63–4.12	0.000
Days from hospital admission to discharge (days) median (IQR)	10 (6–17)	10 (3–17) (1–94)	11 (7–18) (1–75)			0.324
Extraneurological complications associated with complications in the central nervous system (CNS) in patients with COVID-19						
Cardiovascular manifestations de novo, n (%)	43 (4.3)	31 (3.5)	12 (12.5)	3.98	1.97–8.04	0.000
Congestive heart failure de novo, n (%)	20 (2.0)	13 (1.5)	7 (7.3)	5.34	2.08–13.72	0.000
Pulmonary thromboembolism de novo, n (%)	8 (0.8)	5 (0.6)	3 (3.1)	5.74	1.35–24.41	0.034
Deep venous thrombosis de novo, n (%)	11 (1.1)	5 (0.6)	6 (6.3)	11.87	3.55–39.65	0.000
Outcomes						
Invasive mechanical ventilation (IMV), n (%)	310/989 (31.3)	265/893 (29.7)	45/96 (46.9)	2.09	1.36–3.20	0.001
Admission to intensive care unit (ICU), n (%)	146/889 (16.4)	136/801 (17.0)	10/88 (11.4)			0.177
Septic shock, n (%)	277 (28.0)	230 (25.7)	47 (49.0)	2.77	1.80–4.25	0.000
Death, n (%)	433 (43.7)	359 (40.1)	74 (77.1)	5.02	3.06–8.23	0.000

CNS: central nervous system. SOFA: Sepsis Related Organ Failure Assessment. ARDS: acute respiratory distress syndrome. qSOFA: Quick Sepsis Related Organ Failure Assessment. CURB-65: pneumonia prognostic scale, C: confusion, U: urea, R: respiratory rate, B: blood pressure, 65: age > 65 years.

**Table 4:** Mortality predictors in COVID-19 patients with de novo CNS manifestations and complications while in hospital.

Demographic Characteristics and Risk Factors	All Patients n = 991	No Deaths n = 558/991 (56.3%)	Deaths n = 433/991 (43.7%)	CI 95%	*p*	aOR (95%CI)	*p*
≥64 years, n (%)	453 (45.7)	184 (33.0)	269 (62.1)	3.33 (2.57–4.33)	0.000	2.87 (2.02–4.08)	0.000
Male gender, n (%)	614 (62.0)	328 (58.8)	286 (66.1)	1.36 (1.05–1.77)	0.019		
Hypertension, n (%)	549 (55.4)	269 (48.2)	280 (64.7)	1.97 (1.52–2.54)	0.000		
Diabetes mellitus, n (%)	412 (41.6)	205 (36.7)	207 (47.8)	1.58 (1.22–2.04)	0.000		
Chronic kidney disease, n (%)	100 (10.1)	43 (7.7)	57 (13.2)	1.82 (1.20–2.76)	0.005		
Any pulmonary history, n (%)	76 (7.7)	28 (5.0)	48 (11.1)	2.36 (1.45–3.83)	0.000		
COPD, n (%)	50 (5.0)	11 (2.0)	39 (9.0)	4.92 (2.49–9.73)	0.000		
History of heart failure, n (%)	28 (2.8)	9 (1.6)	19 (4.4)	2.80 (1.25–6.25)	0.009		
General neurological (CNS + PNS) manifestations and complications, n (%)	202 (20.3)	97 (17.4)	105 (24.2)	1.52 (1.12–2.08)	0.008		
De novo CNS manifestations and complications while in hospital, n (%)	96 (9.7)	22 (3.9)	74 (17.1)	5.0 (3.06–8.23)	0.000	3.05 (1.39–6.72)	0.006
CNS manifestations							
Delirium, (%)	63 (6.4)	15 (2.7)	48 (11.1)	4.51 (2.49–8.18)	0.000		
Altered consciousness, n (%)	50 (5.0)	6 (1.1)	44 (10.2)	10.41 (4.39–24.66)	0.000		
Drowsiness, n (%)	32/990 (3.2)	7 (1.3)	25/432 (5.8)	4.84 (2.07–11.29)	0.000		
Stupor, n (%)	21 (2.1)	3 (0.5)	18 (4.2)	8.02 (2.35–27.42)	0.000		
Seizures, n (%)	9 (0.9)	4 (0.7)	5 (1.2)		0.515		
Hemiplegia, n (%)	4 (0.4)	1 (0.2)	3 (0.7)		0.324		
Dysarthria, n (%)	5 (0.5)	1 (0.2)	4 (0.9)		0.174		
Aphasia, n (%)	5 (0.5)	0 (0.0)	5 (1.2)		-		
Anisocoria, n (%)	4 (0.4)	0 (0.0)	4 (0.9)		-		
Hallucinations, n (%)	4 (0.4)	1 (0.2)	3 (0.7)		0.324		
CNS syndrome or complications							
Stroke, n (%)	19 (1.9)	3 (0.5)	16 (3.7)	7.09 (2.06–24.52)	0.001		
Ischemic stroke, n (%)	14 (1.4)	3 (0.5)	11 (2.5)	4.82 (1.34–17.39)	0.012		
Ischemic in the left middle cerebral artery, n (%)	7 (0.7)	2 (0.4)	5 (1.2)		0.250		
Ischemic in right middle cerebral artery, n (%)	5 (0.5)	1 (0.2)	4 (0.9)		0.174		
Left carotid ischemic stroke, n (%)	1 (0.1)	0 (0.0)	1 (0.2)		-		
Right carotid ischemic stroke, n (%)	1 (0.1)	0 (0.0)	1 (0.2)		-		
Hemorrhagic stroke, n (%)	5 (0.5)	1 (0.2)	4 (0.9)		0.174		
Subarachnoid hemorrhage	4 (0.4)	0 (0.0)	4 (0.9)		-		
Epilepsy de novo, n (%)	9 (0.9)	4 (0.7)	5 (1.2)		0.515		
Ataxia, n (%)	7 (0.7)	1 (0.2)	6 (1.4)		-		
Encephalitis, n (%)	3 (0.3)	2 (0.4)	1 (0.2)		1.000		
PNS manifestations and complications, n (%)	126 (12.7)	80 (14.3)	46 (10.6)		0.082		
Myalgias, n (%)	185 (18.7)	109 (19.5)	76 (17.6)		0.427		
Dysgeusia, n (%)	94 (9.5)	60 (10.8)	34 (7.9)		0.122		
Anosmia, n (%)	82 (8.3)	58 (10.4)	24 (5.5)		-		
Myopathy, n (%)	4 (0.7)	4 (0.7)	3 (0.7)		1.000		
Guillain Barré syndrome de novo, n (%)	1 (0.1)	1 (0.2)	0 (0.0)		-		
Extraneurological complications associated with complications in patients hospitalized with COVID-19							
Cardiovascular manifestations de novo, n (%)	43 (4.3)	10 (1.8)	33 (7.6)	4.52 (2.20–9.28)	0.000		
Laboratory findings							
Lymphopenia ≤ 0.900 (10^3^/µL), n (%)	386/978 (39.5)	189/553 (34.2)	197/425 (46.4)	1.66 (1.28–2.16)	0.000		
Platelets ≤ 150 (10^3^/µL), n (%)	137/982 (14.0)	53/553 (9.6)	84/429 (19.6)	2.30 (1.59–3.33)	0.000		
D-dimer ≥ 1000 ng/mL, n (%)	361/912 (39.6)	141/522 (27.0)	220/390 (56.4)	3.49 (2.65–4.62)	0.000	2.25 (1.57–3.23)	0.000
CORADS 6, n (%)	141/803 (17.6)	60/461 (13.0)	81/342 (23.7)	2.07 (1.44–2.99)	0.000		
Severe ARDS, n (%) on admission	301/902 (33.4)	59/473 (12.5)	242/429 (56.4)	9.08 (6.51–12.67)	0.000		
SOFA ≥ 5, n (%)	191/702 (27.2)	39/349 (11.2)	152/353 (43.1)	6.01 (4.05–8.91)	0.000	4.52 (2.93–6.99)	0.000
NEWS2 score ≥ 8, n (%)	313/874 (35.8)	120/469 (25.6)	193/405 (47.7)	2.65 (1.99–3.52)	0.000		
Outcomes							
IMV, n (%)	310/989 (31.3)	32 (5.7)	278/431 (64.5)	29.87 (19.87–44.90)	0.000		
Intensive care unit (ICU), n (%)	146/889 (16.4)	51/469 (10.9)	95/420 (22.6)	2.39 (1.66–3.47)	0.000		

CNS: central nervous system. PNS: peripheral nervous system. COPD: chronic obstructive pulmonary disease. ARDS: acute respiratory distress syndrome. SOFA: sepsis related organ failure assessment. ng/mL: nanograms/milliliters. IMV: invasive mechanical ventilation.

## Data Availability

Not applicable.
